# Author Correction: Architectural basis for cylindrical self-assembly governing Plk4-mediated centriole duplication in human cells

**DOI:** 10.1038/s42003-023-05162-w

**Published:** 2023-07-27

**Authors:** Jong Il Ahn, Liang Zhang, Harsha Ravishankar, Lixin Fan, Klara Kirsch, Yan Zeng, Lingjun Meng, Jung-Eun Park, Hye-Yeoung Yun, Rodolfo Ghirlando, Buyong Ma, David Ball, Bonsu Ku, Ruth Nussinov, Jeremy D. Schmit, William F. Heinz, Seung Jun Kim, Tatiana Karpova, Yun-Xing Wang, Kyung S. Lee

**Affiliations:** 1 Cancer Innovation Laboratory, Center for Cancer Research, National Cancer Institute, National Institutes of Health, Bethesda, MD 20892 USA; 2 Basic Science Program, Frederick National Laboratory for Cancer Research, Small-Angle X-ray Scattering Core Facility, National Cancer Institute, National Institutes of Health, Frederick, MD 21702 USA; 3 Disease Target Structure Research Center, Korea Research Institute of Bioscience and Biotechnology, Daejeon, Republic of Korea; 4 Laboratory of Molecular Biology, National Institute of Diabetes and Digestive and Kidney Diseases, National Institutes of Health, Bethesda, MD 20892 USA; 5 Basic Science Program, Leidos Biomedical Research, Inc., Cancer and Inflammation Program, National Cancer Institute, Frederick, MD 21702 USA; 6 Laboratory of Receptor Biology and Gene Expression, Optical Microscopy Core, National Cancer Institute, National Institutes of Health, Bethesda, MD 20892 USA; 7 Department of Human Molecular Genetics and Biochemistry, Sackler School of Medicine, Tel Aviv University, Tel Aviv, 69978 Israel; 8 Department of Physics, Kansas State University, Manhattan, KS 66506 USA; 9 Optical Microscopy and Analysis Laboratory, Cancer Research Technology Program, Frederick National Laboratory for Cancer Research, Frederick, MD 21702 USA; 10 Protein-Nucleic Acid Interaction Section, Center for Structural Biology, National Cancer Institute, National Institutes of Health, Frederick, MD 21702 USA; 11 Present Address: School of Pharmacy, Shanghai Jiao Tong University, 200240 Shanghai, China

**Keywords:** Cell growth, Biophysical chemistry

Correction to: *Communications Biology* 10.1038/s42003-023-05067-8, Article published online 11 July 2023

In the original version of the Article, Figure 3 contained misaligned y-axis labels for panels 3a and 3b.

Previous Figure 3:
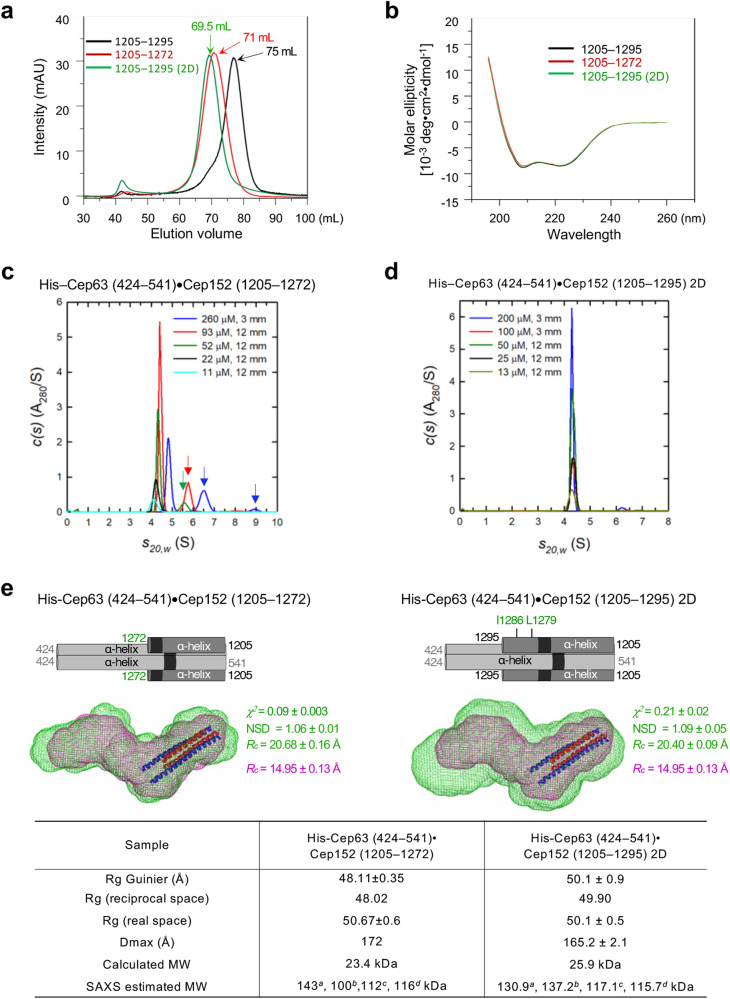


Corrected Figure 3:
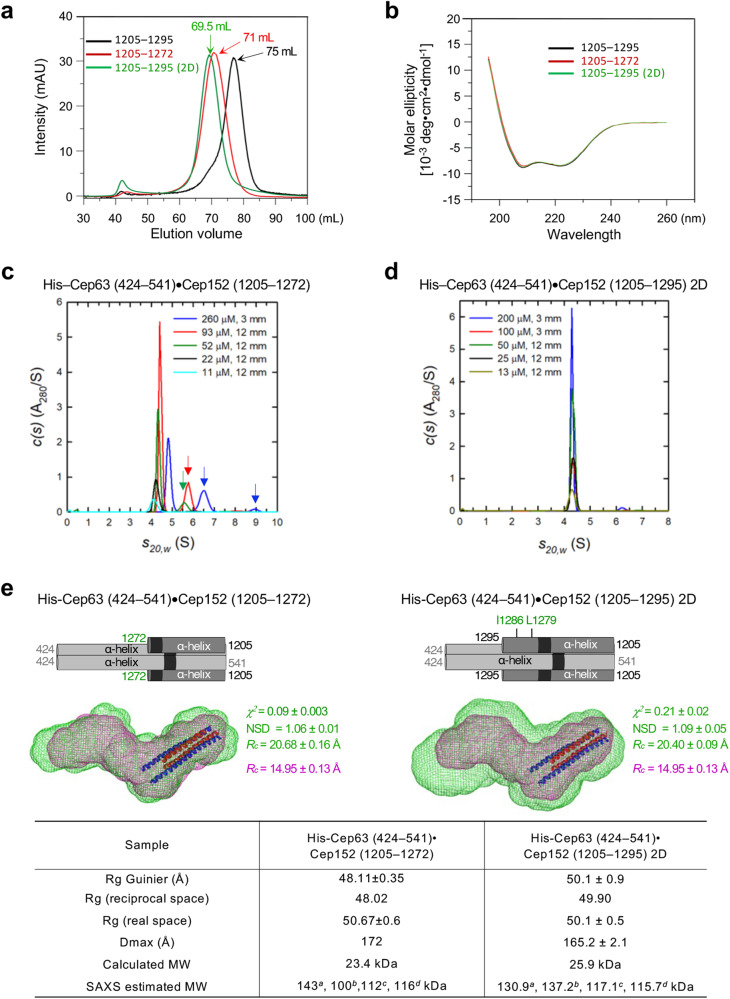


This has been corrected in the PDF and HTML versions.

