# Supplementary material for: Architectural basis for cylindrical self-assembly governing Plk4-mediated centriole duplication in human cells
Source: Commun Biol. 2023 Jul 11;6:712. doi: 10.1038/s42003-023-05067-8 (PMC10336005; doi:10.1038/s42003-023-05067-8)
Supplement: Supplementary file 2 — Supplementary Information [file 42003_2023_5067_MOESM2_ESM.pdf]

Supplementary Information for

Architectural basis for cylindrical self-assembly governing Plk4-mediated centriole duplication in human cells

Jong Il Ahn, Liang Zhang, Harsha Ravishankar, Lixin Fan, Klara Kirsch, Yan Zeng, Lingjun Meng, Jung-Eun Park, Hye-Yeoung Yun, Rodolfo Ghirlando, Buyong Ma, David Ball, Bonsu Ku, Ruth Nussinov, Jeremy D. Schmit, William F. Heinz, Seung Jun Kim, Tatiana Karpova, Yun-Xing Wang, Kyung S. Lee

This file includes:

Supplementary Figures 1–8

Supplementary References

Supplementary Tables 1–4

Supplementary Movies 1–3

Supplementary Fig. 1

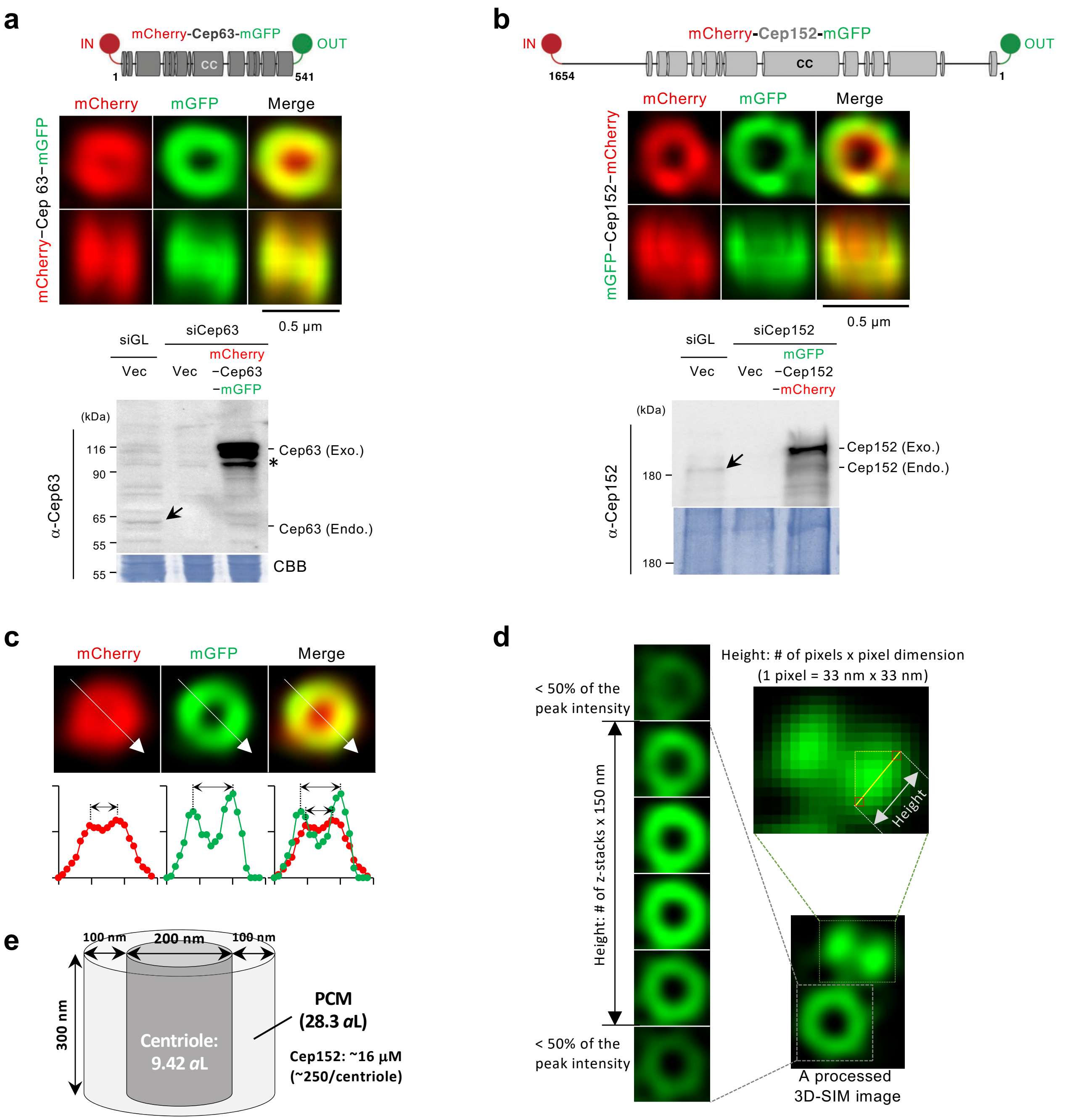

Supplementary Fig. 1. 3D-SIM images and methods used for determining the diameters and heights of cylindrically localized Cep63 and Cep152 signals. **a, b** Fluorescence images used for generating surface-rendered models in Fig. 1a. Immunoblots (bottom) show the lentiviral expression of exogenous (Exo.) mCherry-Cep63-mGFP or mGFP-Cep152-mCherry in comparison to the level of their respective endogenous (Endo.) protein. Arrows, the expected exogenous proteins expressed; asterisk, degradation product; CBB, Coomassie Brilliant Blue-stained membrane. **c** Line-scan plots showing how the peak-to-peak diameters (double arrows) of the mCherry and mGFP fluorescence were measured. **d** Procedures taken to measure the height of the Cep63 and Cep152 cylindrical signals. Signals less than 50% of the peak fluorescence intensity were excluded from the height calculation. **e** A schematic showing the inner PCM space and an expected concentration of Cep152, estimated to be 540 molecules per centrosome [1].

Supplementary Fig. 2

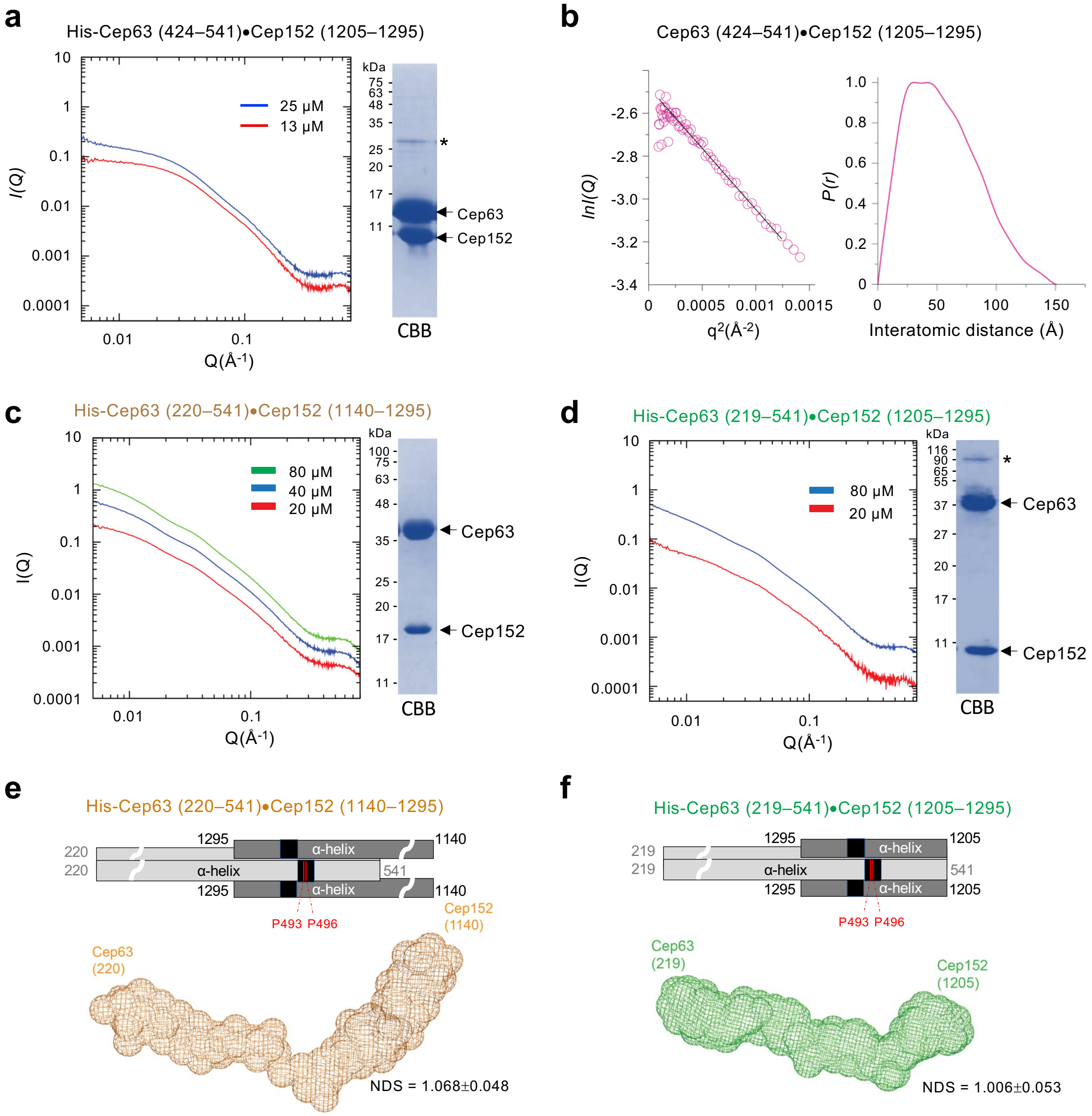

Sample	His-Cep63 (220–541) •Cep152 (1140–1295)	His-Cep63 (219–541) •Cep152 (1205–1295)
Rg Guinier (Å)	104 ± 8	86 ± 12
Rg in reciprocal space	102.2	85
Rg in real space	103 ± 3	85 ± 2
Dmax (Å)	338 ± 20	287 ± 20
Calculated MW	56.1 kDa	49.2 kDa
SAXS estimated MW	106[a], 101.7[b], 103[c], 94.6[d] kDa	119[a], 94[b], 90.9[c], 81.8[d] kDa

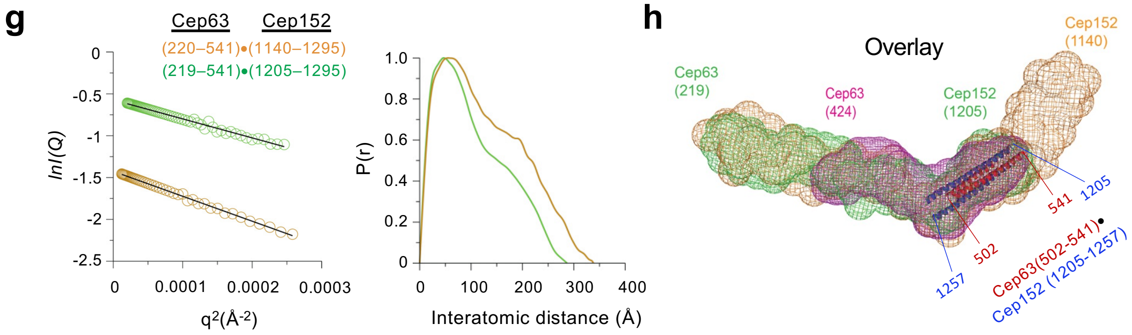

Supplementary Fig. 2

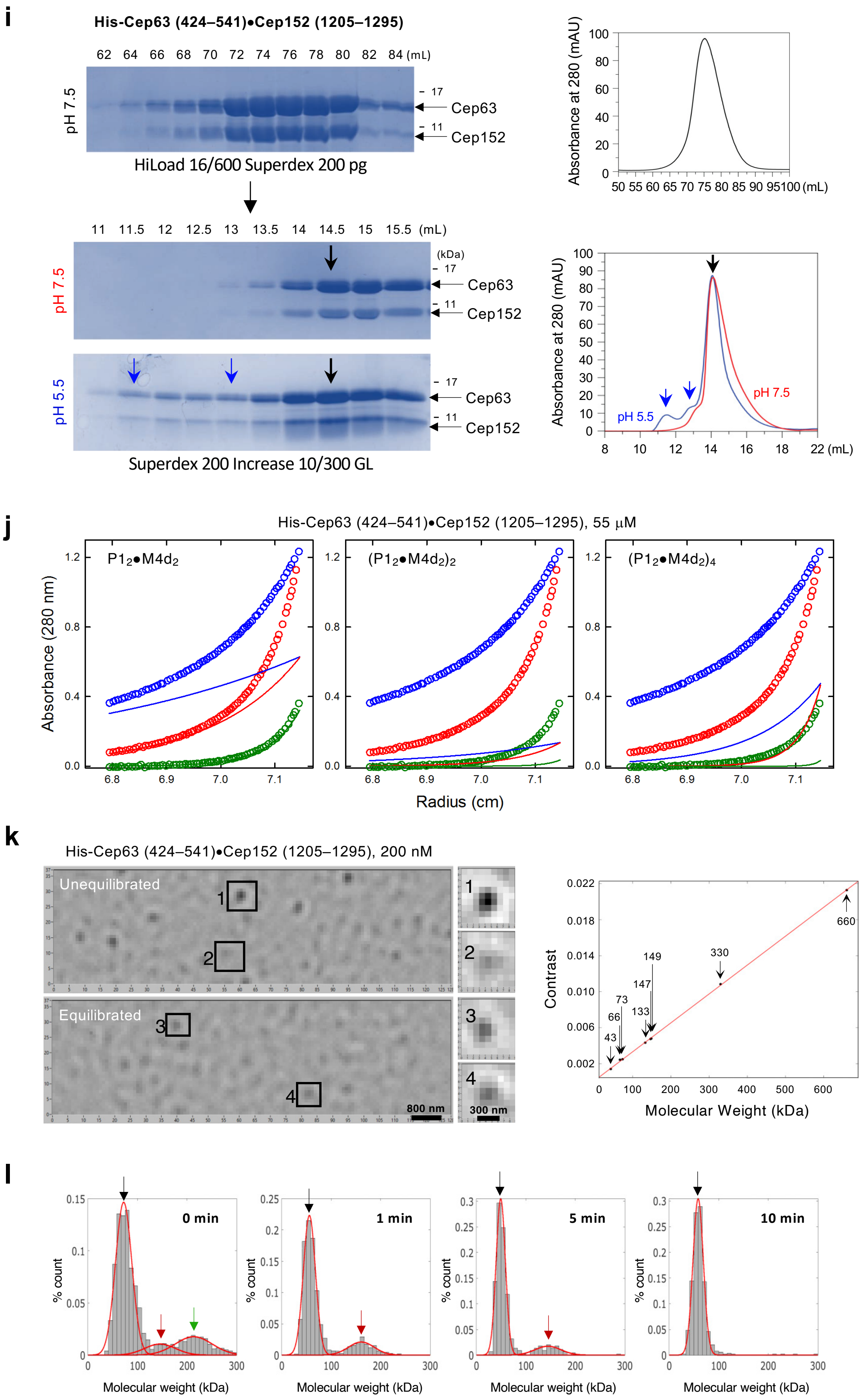

Supplementary Fig. 2. Detection of concentration-dependent formation of higher-MW Cep63•Cep152 complexes. **a** The SAXS intensity profiles of the Cep63 (424–541)•Cep152 (1205–1295) complex were analyzed at the concentrations indicated. The protein separated by SDS-PAGE is stained with Coomassie Brilliant Blue (CBB) (right). Asterisk, contaminating protein. **b** Guinier analysis (left) and pairwise distribution function of interatomic vector *P(r)* curves (right) from the extrapolated (at infinite dilution) SAXS data of the complex in (**a**). The linear Guinier fit is shown as a black line. **c**–**h** SAXS measurement and analysis for the Cep63 (220–541)•Cep152 (1140–1295) and Cep63 (219–541)•Cep152 (1205–1295) protein complexes. The SAXS intensity profiles at various concentrations and the SDS-PAGE gels of the indicated complexes (**c, d**) were analyzed as in Fig. 2a and Supplementary Fig. 2b. Asterisk, contaminating protein. (**e**–**g**) Schematic diagrams for the indicated complexes and the physical parameters calculated from their respective SAXS data (**c, d**) are provided. (**h**) Overlay of DAMMIN generated ab-initio shape envelopes is derived from SAXS data from (**a**, **c**, **d**). 3D-rendered envelopes and their overlay are provided in Supplementary Movie 2. **i** SEC profiles and corresponding CBB-stained gels of the Cep63 (424–541)•Cep152 (1205–1295) complex purified with the HiLoad 16/600 Superdex 200 pg column at pH 7.5 (top) and subsequently analyzed with the Superdex 200 Increase 10/300 GL column (bottom) at pH 7.5 and pH 5.5. Black arrow, the tetrameric Cep63 (424–541)•Cep152 (1205–1295) complex with the calculated MW of 51.8 kDa (Fig. 2c). Higher-MW complexes (blue arrows) appear at pH 5.5. **j** Sedimentation equilibrium absorbance data for the Cep63 (424–541)•Cep152 (1205–1295) complex, collected at 55 μM, pH 5.5, showing the modeled contributions (indicated as solid lines) of the tetramer (left), octamer (center), and hexadecamer (right). The contribution of each species to the overall sedimentation equilibrium profile is shown for each of the rotor speeds: 7,000 (blue), 11,000 (red), and 20,000 (green) rpm. **k** Representative differential interferometric scattering images for the Cep63 (424–541)•Cep152 (1205–1295) complex analyzed at 200 nM, pH 5.5, taken immediately after diluting the protein from the 200 μM stock (unequilibrated, top) or after diluting it to 200 nM and letting it equilibrate for 2 hours at RT (equilibrated, bottom). Boxes, areas of enlargement. The graph (right) shows contrast versus MW for the proteins used for mass calibration—BSA monomer (66 kDa) and dimer (133 kDa), ADH dimer (73 kDa) and tetramer (147 kDa), ovalbumin (43 kDa), thyroglobulin monomer (330 kDa) and dimer (660 kDa), and anti-human thrombin antibody (149 kDa). **l** iSCAMS analyses carried out at the indicated time points after diluting the same sample in (**e**) to 100 nM at pH 5.5. Percentages of particle counts (Incidences) are shown as a function of time. Black arrow, heterotetramer; red arrow, octamer; green arrow, hexadecamer.

Supplementary Fig. 3

a

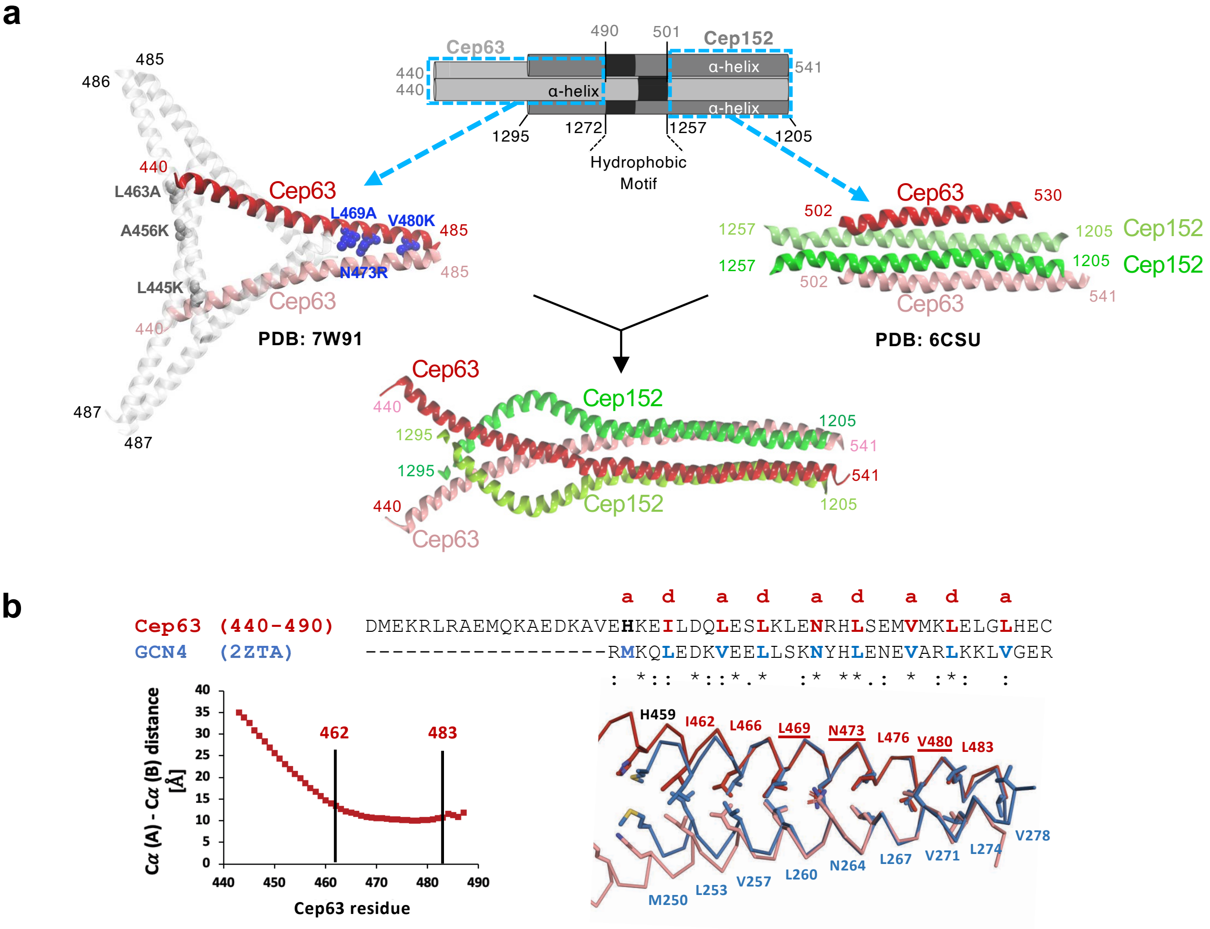

c

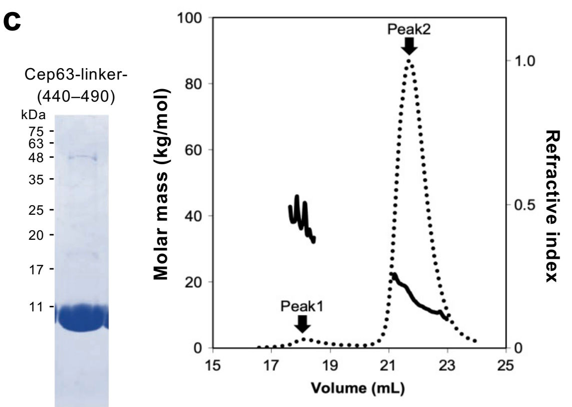

Cep63-linker (440–490)	SEC-MALS MW (kDa)	Mass fraction (%)	Polydispersity (Mw/Mn)
Peak1	39.2 (±13.3%)	2.1%	1.037 (±17.98%)
Peak2	15.2 (±9.81%)	97.9%	1.064 (±14.02%)

d

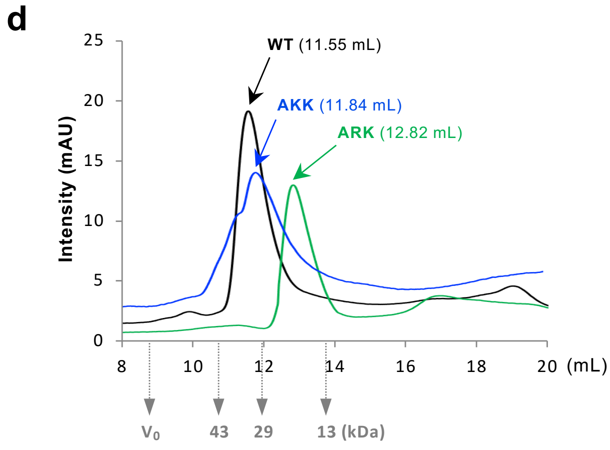

e

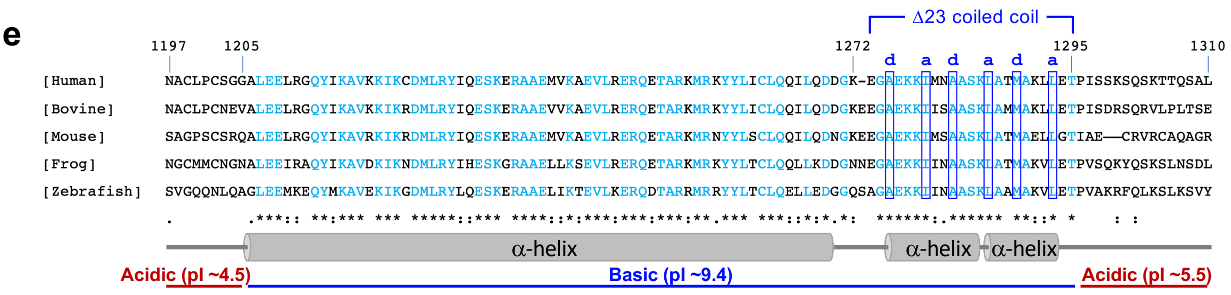

Supplementary Fig. 3

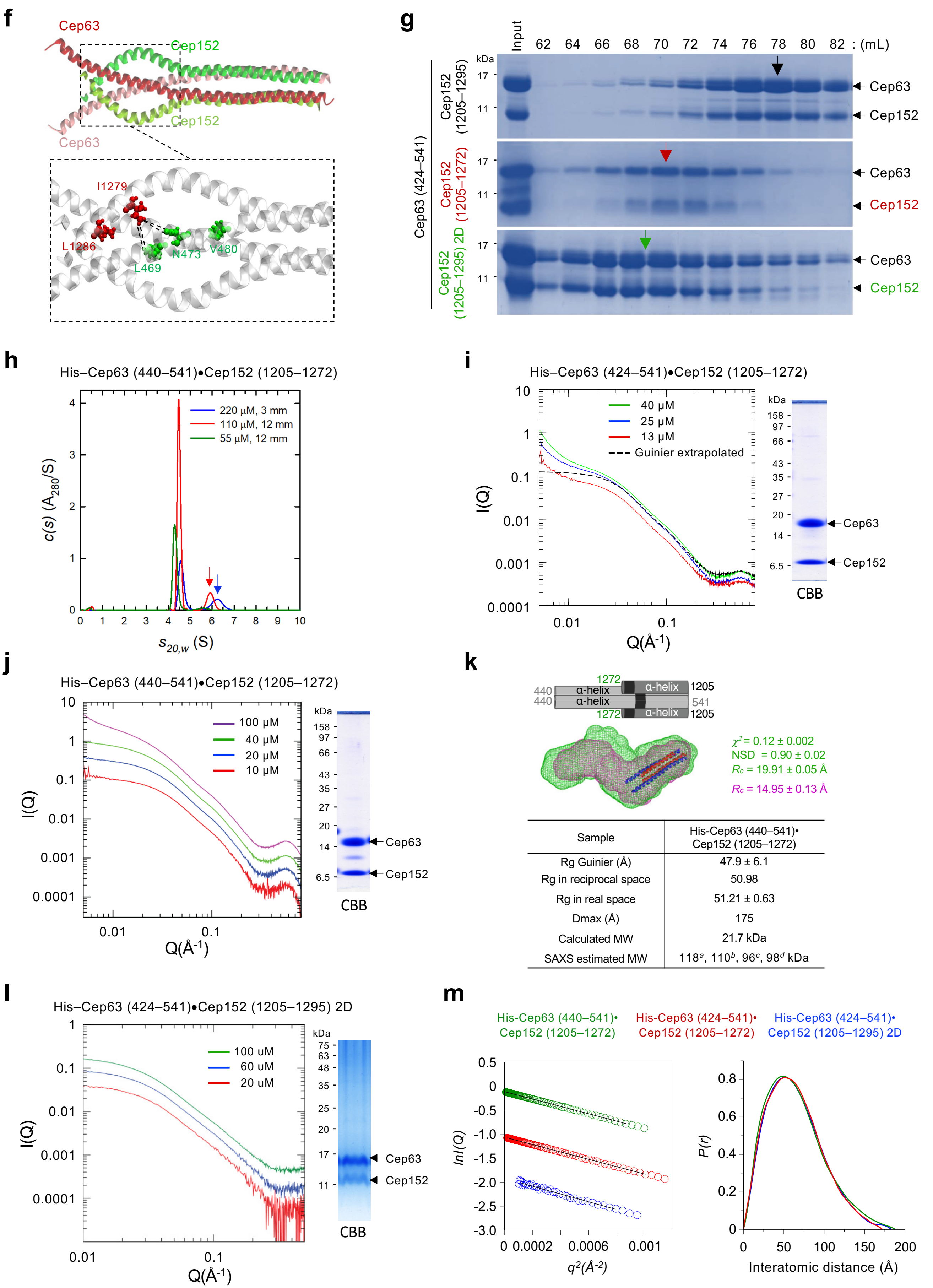

Sample	His-Cep63 (440–541)• Cep152 (1205–1272)
Rg Guinier (Å)	47.9 ± 6.1
Rg in reciprocal space	50.98
Rg in real space	51.21 ± 0.63
Dmax (Å)	175
Calculated MW	21.7 kDa
SAXS estimated MW	118[a], 110[b], 96[c], 98[d] kDa

Supplementary Fig. 3. The molecular nature of the Cep63 (424–541)•Cep152 (1205–1295) complex and requirement of the basic Cep152 (1273–1295) CC motif for the structural integrity of the complex. **a** Schematic showing the Cep63 (424–541)•Cep152 (1205–1295) complex and the fragments (dotted boxes, cyan) used to determine the X-ray crystal structures below. The crystal structure of the Cep63 (502–541)•Cep152 (1205–1250) four-helix bundle at right (PDB: 6CSU) has been reported [2]. The dimeric Cep63 (440–485) structure (PDB: 7W91; Supplementary Table 4) present in the hexameric asymmetric unit was modeled as a part of the Cep63 (424–541)•Cep152 (1205–1295) heterotetramer. The model for the entire complex was built by homology modeling through the process of merging a dimer of the hexameric asymmetric unit (left) and the four-helix bundle (right) (PDB: 6CSU) (see Methods for details). The residues important for forming the Cep63 (440–485) dimer (i.e., L469, N473, V480) and the residues involved in the antiparallel inter-dimeric interactions (i.e., L445, A456, L463) are marked. The interdimeric interactions appeared to be generated through crystal packing (see text for details). **b** The Cep63 (440–490) region forms a dimer via leucine-zipper-like interactions. (Top) Primary sequence comparison between Cep63 (440–490) and the leucine zipper motif of GCN4 (PDB: 2ZTA), with the hydrophobic residues indicated in red (the **a** and **d** positions in the helical wheel). (Bottom) Overlay of the structures of the Cep63 dimer (PDB: 7W91) (red) with that of the GCN4 dimer (PDB: 2ZTA) (blue), showing pairwise hydrophobic interactions. The residues upstream of Cep63 I462 are moving away from each other (left graph). **c** SEC-MALS analysis demonstrating the Cep63 (440–490) dimer (peak 2) with a small fraction (2.1%) of a higher-MW species. The CBB-stained gel (left) shows the protein used for the analysis. **d** SEC profiles showing that the L469A, N473R, V480K (ARK) mutations, but not the L445A, A456K, L463K (AKK) mutations, disrupt the homodimeric interactions in Cep63 (440–490), suggesting that the Cep63 (440–490) dimer arranged in a pairwise manner [shown in red in (**a**)] is the primary form. The monomeric Cep63-linker-(440–541) is calculated to be 8.1 kDa due to the presence of a linker immediately upstream of the D440 residue. **e** Multiple sequence alignment for human Cep152 transcription variant 2 (residues 1–1654) (GenBank accession number: NP_055800.2) and its orthologs, performed using the Clustal Omega software. The primary amino acid sequences of Cep152 orthologs are human Cep152 tv1 (NCBI RefSeq: NP_001181927.1), human Cep152 tv2 (NCBI RefSeq: NP_055800.2), cow Cep152 (UniProtKB: E1BCR3), mouse Cep152 (UniProtKB: A2AUM9), frog Cep152 (UniProtKB: Q498G2), and zebrafish Cep152 (UniProtKB: X1WBK2). (*), positions with a single and fully conserved amino acid residue; (:), positions with amino acid residues conserved between groups of strong similar properties; (.), positions with amino acid residues conserved between groups of weakly similar properties. Letters in cyan, identical residues; gray bar, α-helix as predicted by the PSIPRED server; Δ23, deletion of the basic coiled-coil motif made of 23 residues (1273–1295). The **a** and **d** positions of the helical wheel in the putative Cep152 (1273–1295) are indicated in blue. **f** A Cep63 (440–541)•Cep152 (1205–1295) structure model showing I1279 and L1286 residues in Cep152 and their interacting residues in Cep63. **g** CBB-stained gels of the indicated complexes fractionated by SEC. Arrows, the peak position of the respective proteins. **h** Sedimentation velocity *c(s)* profiles for the Cep63 (440–541)•Cep152 (1205–1272) truncation mutant at various loading concentrations. The slow-sedimenting 4.24 S complex observed at low concentrations has an estimated MW of 64 kDa (the best-fit frictional ratio of 1.32), suggesting that it forms a putative hexamer. Arrows, higher-MW complexes and/or aggregates observed at high concentrations. **i**. SAXS scattering profiles of the Cep63 (424–541)•Cep152 (1205–1272) truncation (shown in the CBB-stained gel). The Guinier extrapolated data at infinite dilution are shown as a dashed black line. **j, k** SAXS scattering profiles for the Cep63 (440–541)•Cep152 (1205–1272) complex (shown in the CBB-stained gel) (**j**) and a calculated envelope of the complex (green) overlaid with that of the Cep63 (424–541)•Cep152 (1205–1295) complex (Fig. 2a) (**k**). The χ^2, NSD, and *Rc* values were calculated as in Fig. 3e. Physical parameters from the SAXS data of the complexes are also provided (table). **l** SAXS scattering data for the Cep63 (424–541)•Cep152 (1205–1295) 2D mutant complex (shown in the CBB-stained gel) obtained at the indicated concentrations. **m** Guinier analyses (left) and pairwise distribution functions of interatomic distances (right) calculated from the SAXS data of the complexes in (**i**, **j**, **l**) extrapolated to zero concentration. Their respective Guinier fit lines are provided (left, black lines).

Supplementary Fig. 4

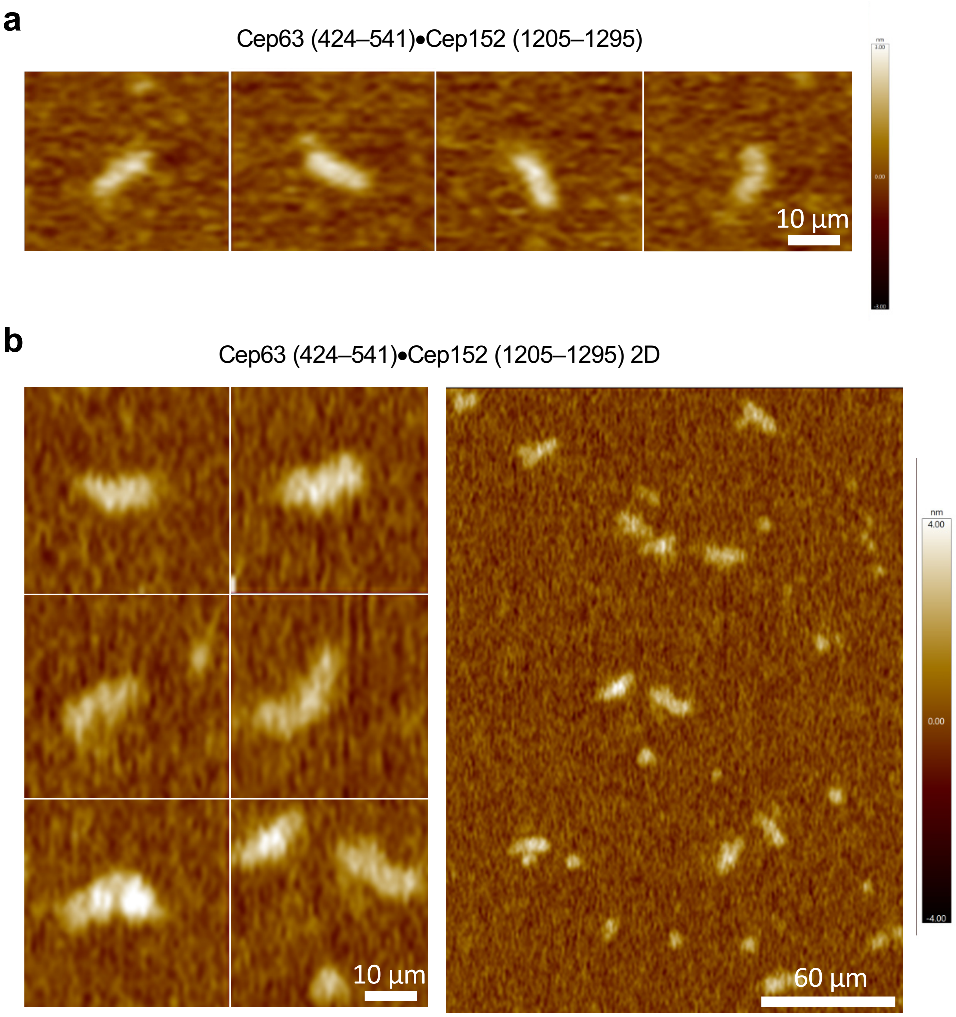

Supplementary Fig. 4. Visualization of the Cep63•Cep152 complex by AFM. Representative raw images of the His-Cep63 (424–541)•Cep152 (1205–1295) (**a**) and His-Cep63 (424–541)•Cep152 (1205–1295) 2D (**b**) complexes are shown. Images of the complexes were acquired by tapping mode AFM imaging under physiological buffer conditions [20 mM Tris-Cl (pH 7.8), 150 mM NaCl, 1 mM TCEP] at 5 ºC. All images shown are unprocessed raw images.

Supplementary Fig. 5

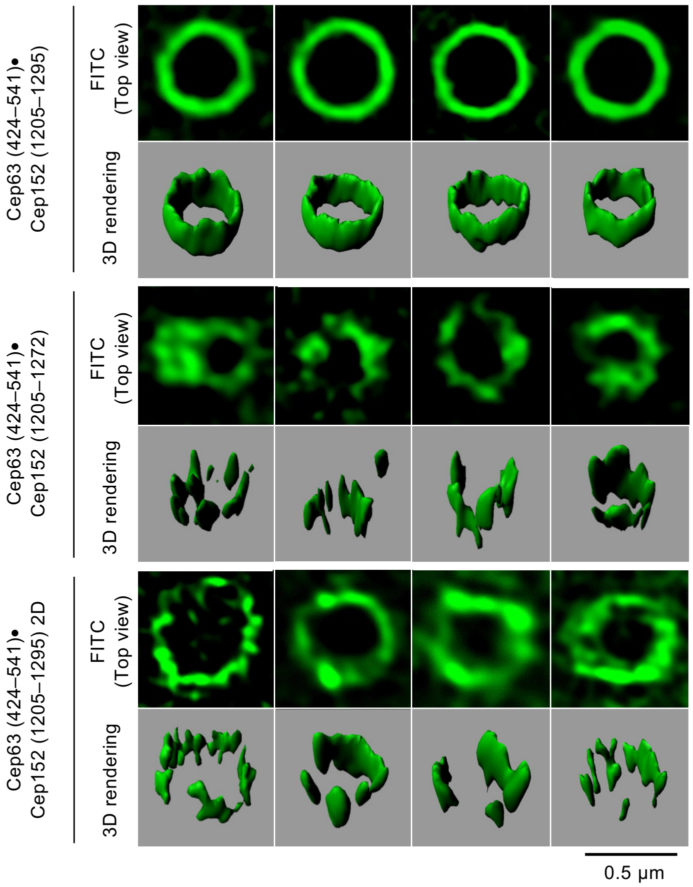

Supplementary Fig. 5. Representative examples of 3D-SIM (top) and surface-rendered (bottom) images for the *in vitro* self-assemblies generated by the same protein complexes used for Fig. 5. Bars, 500 nm. The Cep63 (424–541)•Cep152 (1205–1272) truncation complex lacking the basic CC (1273–1295) residues (i.e., Δ23) generated mostly incomplete self-assemblies or aggregates, while the Cep63 (424–541)•Cep152 (1205–1295) (I1279D, L1286D) (i.e., 2D) mutant formed highly disorganized cylinders with patches of fluorescence signals. Quantified data from three independent experiments are provided in Fig. 5b, c.

Supplementary Fig. 6

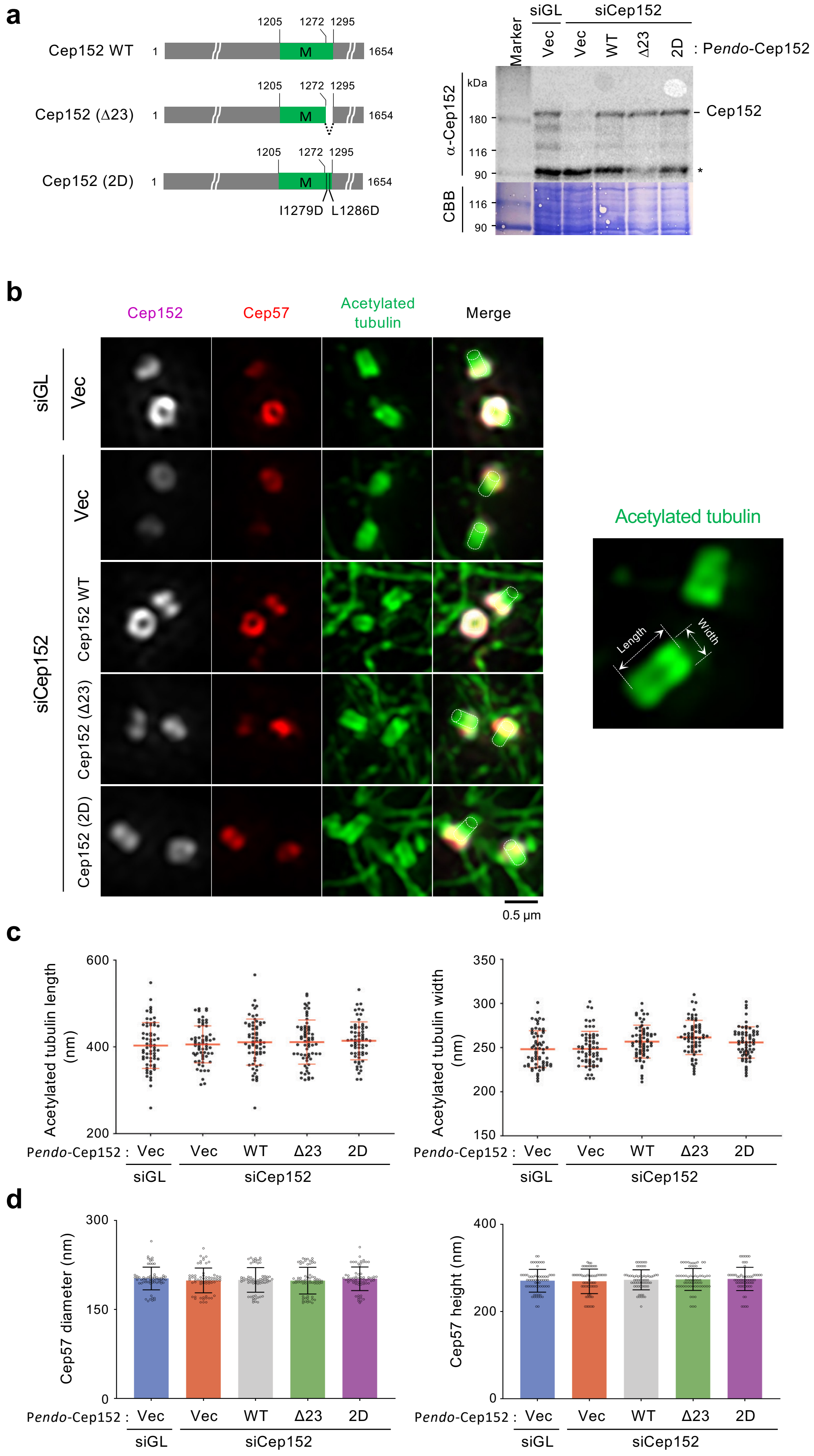

Supplementary Fig. 6

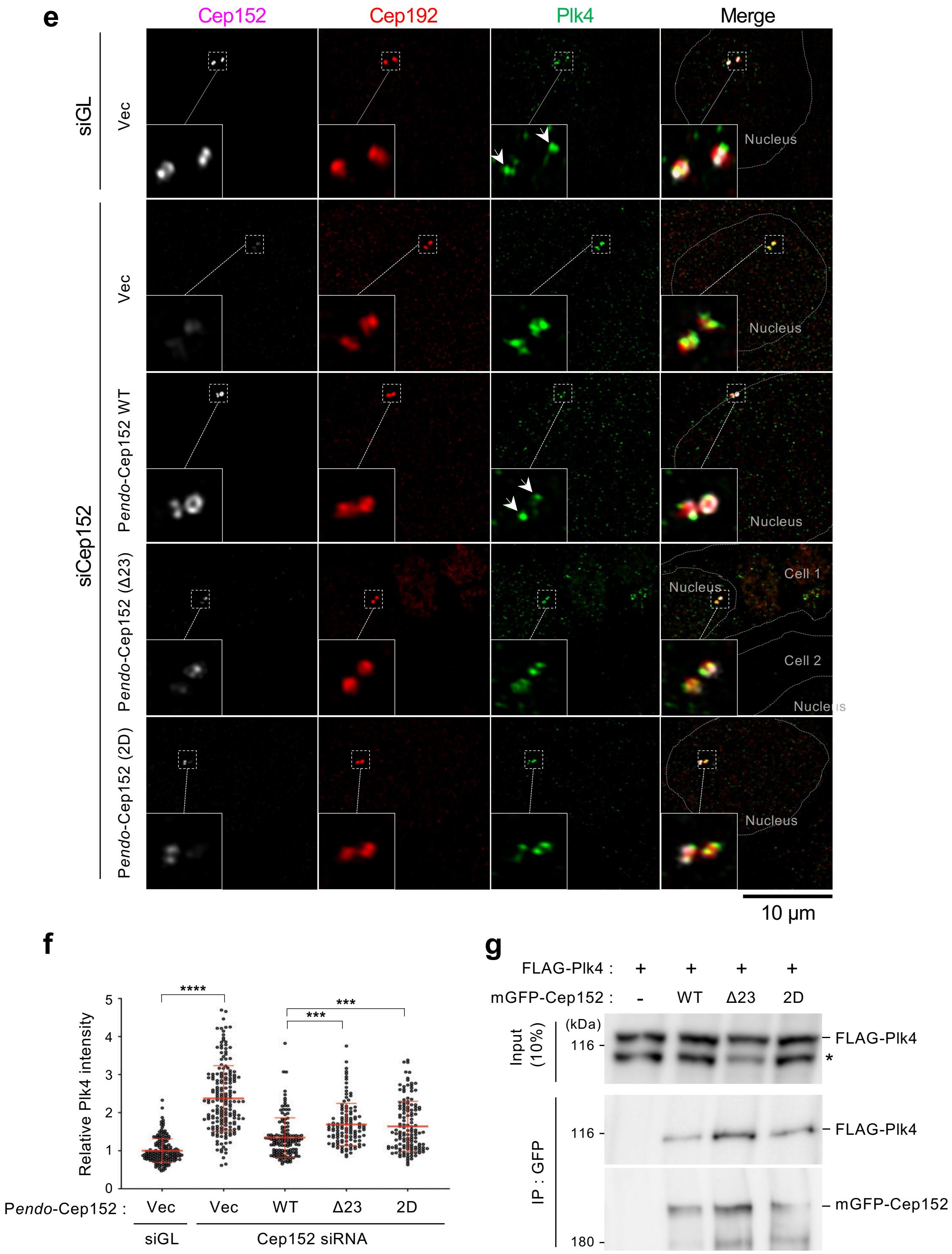

Supplementary Fig. 6

h

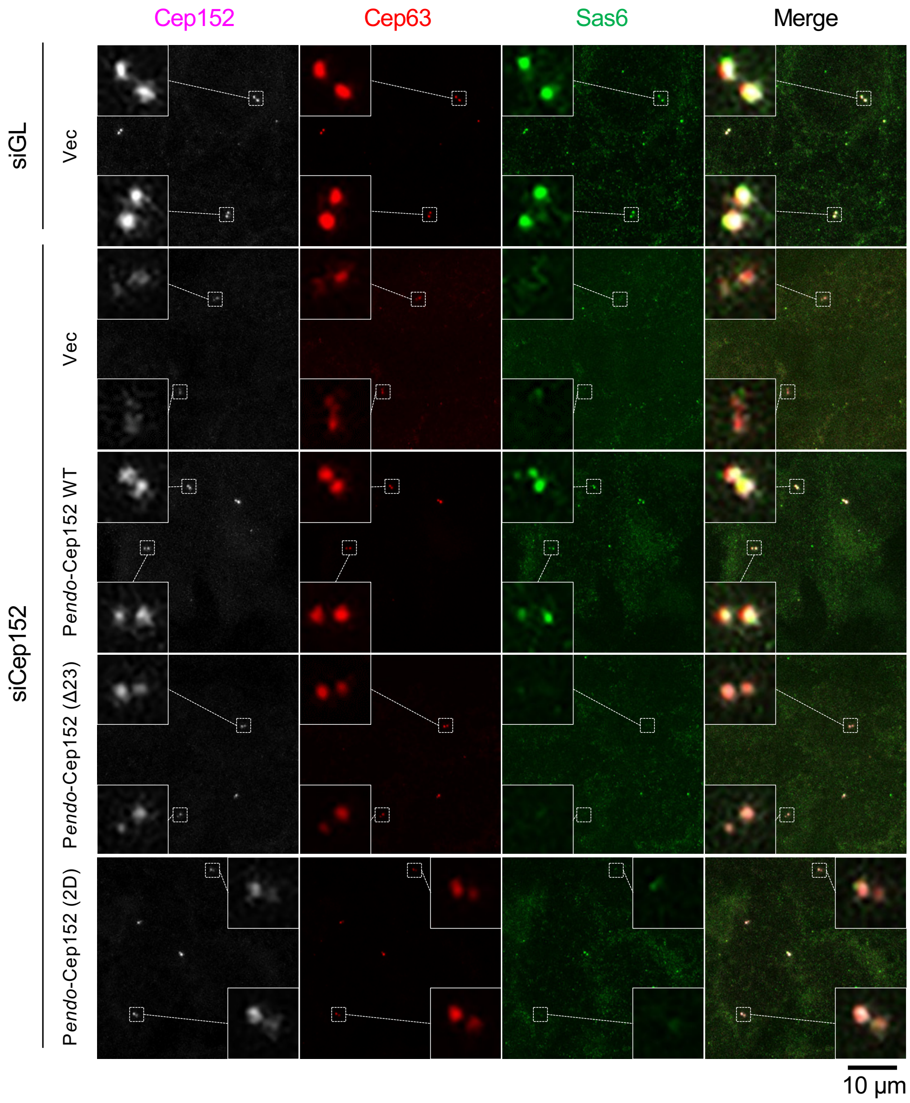

i

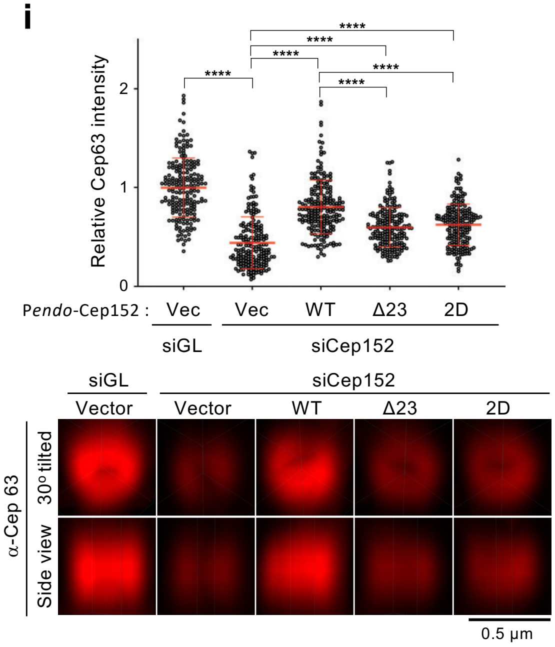

j

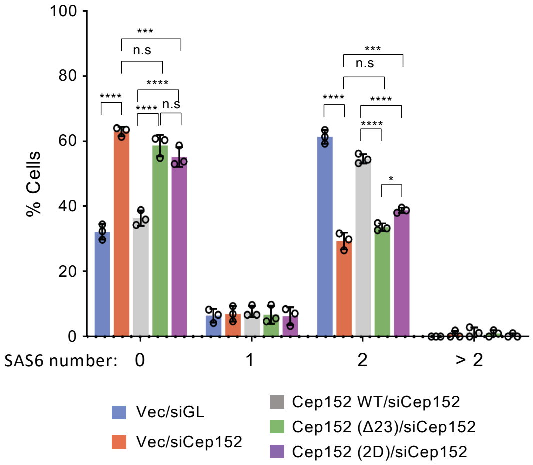

Supplementary Fig. 6

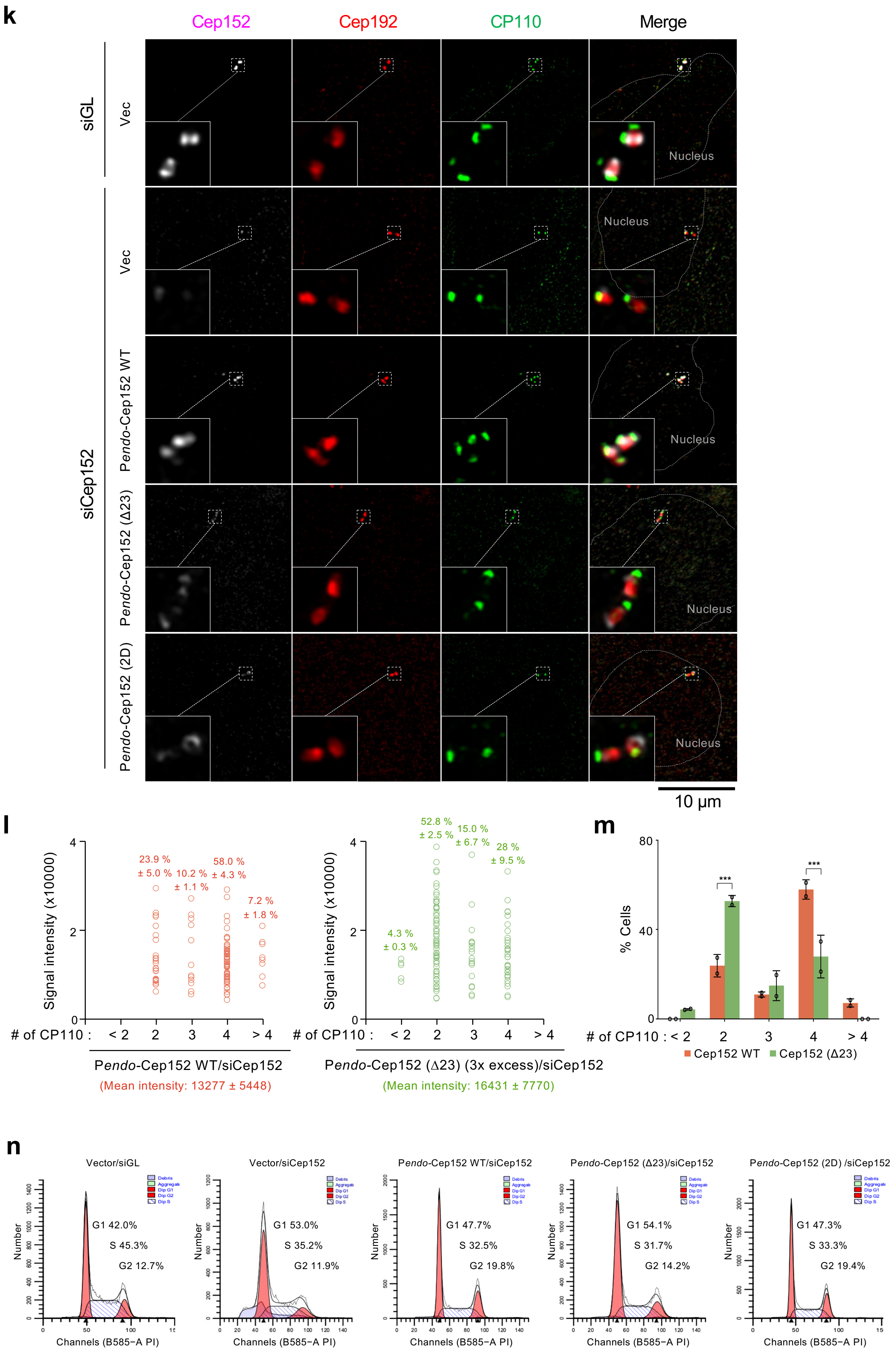

Supplementary Fig. 6. The conserved basic CC motif of Cep152 is required for proper centriole duplication. **a–d** U2OS cells stably expressing endogenous promoter (P*endo*)-controlled siCep152-insensitive constructs (diagram, left) were immunoblotted (**a**), immunostained with the indicated antibodies after depleting endogenous Cep152 (**b**), and quantified (**c, d**). The 1205–1295 region (marked "M") and the location of the 1273–1295 deletion (i.e., Δ23) and the I1279D, L1286D (i.e., 2D) mutations are indicated in (**a**, left). The immunoblotting results (**a**, right) show the level of Cep152 expression for the cells used here and in Fig. 6 (right). Asterisk, a cross-reacting protein; CBB, Coomassie Brilliant Blue–stained membrane. Representative images and methods used to quantify acetylated tubulin signals are shown in (**b**). Quantified data in (**c, d**) were obtained from a total of n = 65 for each sample. Bars, mean of n ± s.d. **e**, **f** Images from 3D-SIM (**e**) and quantified Plk4 signal intensities from confocal microscopy (**f**) were obtained after immunostaining the cells in (**a**) with the indicated antibodies. Box, area of enlargement; arrows, dot-state Plk4. Ring-state versus dot-state Plk4 in (**e**) was quantified from three independent experiments, and the result is shown in Fig. 6e. Quantification of confocal-imaged Plk4 intensities (**f**) was performed from three independent experiments [per experiment, n ≥ 42 for Vec/siGL (total n = 131); n ≥ 43 for Vec/siCep152 (total n = 138); n ≥ 39 for Cep152/siCep152 (total n = 132); n ≥ 37 for Cep152 (Δ23)/siCep152 (total n = 118), n ≥ 43 for Cep152 (2D)/siCep152 (total n = 136)]. ***$P < 0.001$, ****$P < 0.0001$ (unpaired two-tailed *t*-test). Bars, mean of n ± s.d. **g** Coimmunoprecipitation analysis performed with HEK293T cells transfected with the indicated constructs. Asterisk, a degradation product. **h–j** 3D-SIM imaging (**h**), quantification of Cep63 signal intensities (top) and representative Cep63 images (bottom) (**i**), and quantification of Sas6 signals (**j**) were performed similarly as in (**e, f**). Quantification was performed from three independent experiments [per experiment: for Cep63 signal intensities (**i**), n ≥ 65 for Vec/siGL (total n = 202); n ≥ 60 for Vec/siCep152 (total n = 201); n ≥ 68 for Cep152/siCep152 (total n = 210); n ≥ 59 for Cep152 (Δ23)/siCep152 (total n = 203), n ≥61 for Cep152 (2D)/siCep152 (total n = 208); for Sas6 counts (**j**), n ≥ 104 for Vec/siGL (total n = 331); n ≥ 64 for Vec/siCep152 (total n = 272); n ≥ 90 for Cep152/siCep152 (total n = 298); n ≥ 102 for Cep152 (Δ23)/siCep152 (total n = 315), n ≥98 for Cep152 (2D)/siCep152 (total n = 327). *$P < 0.05$, ***$P < 0.001$, ****$P < 0.0001$ (unpaired two-tailed *t*-test). Bars, mean of three experiments ± s.d. **k–m** 3D-SIM imaging and quantification of the cells in (**a**) after immunostaining with the indicated antibodies. The number of CP110 dots in (**k**) was counted from three independent experiments, and the results are provided in Fig. 6f. Box, area of enlargement. (**l, m**) To examine whether restoring the pericentriolar Cep152 (Δ23) mutant to the level of the corresponding Cep152 WT rescues the impaired CP110 recruitment in these cells (Fig. 6f), U2OS cells transfected with P*endo*-Cep152 WT or P*endo*-Cep152 (Δ23) (3-fold excess amount) were immunostained and analyzed from two independent experiments. Data quantified according to Cep152 intensities and CP110 dot numbers are shown (**l**). Per experiment, n ≥ 51 for Cep152/siCep152 (total n = 110) and n ≥ 49 for Cep152 (Δ23)/siCep152 (total n = 115). ***$P < 0.001$ (unpaired two-tailed *t*-test). Note that even under these conditions with similar levels of centrosome-associated Cep152 WT and Cep152 (Δ23), the Cep152 (Δ23)-expressing cells exhibit a much-reduced level of four CP110 dot-containing cells (28.2%) when compared with the Cep152 WT-expressing cells (61%). The graph in (**m**) was generated by replotting the same data in (**l**) to show a significantly reduced level of CP110 dot signals in the Cep152 (Δ23) mutant-expressing cells. **n** Flow cytometry analyses were performed for the cells in (**a**) and acquired data were analyzed by the Modfit LT software (through manual analyses with auto linearity and a gate for doublet discrimination; gating images are shown in Supplementary Fig. 8). Note that Cep152 RNAi cells expressing Cep152 (2D) did not show any apparent delay in the cell cycle, whereas the cells expressing Cep152 (Δ23) exhibited a delay to a degree similar to that of the control vector-expressing cells (as a result, the 4N-state population decreased by 6.4%). However, even after normalizing the S/G2 population to the level of the Cep152 WT-expressing cells, the Cep152 (Δ23)-expressing cells still showed a statistically significant level (****$P < 0.0001$, unpaired two-tailed *t*-test) of centriole biogenesis defect. Only the siCep152 cells expressing vector (i.e., Vector/siCep152) show a significant dying population indicated by the sub-G1 "debris." X-axis, Propidium iodide (PI) intensity.

a

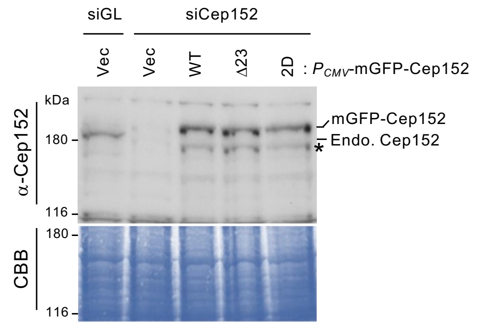

b

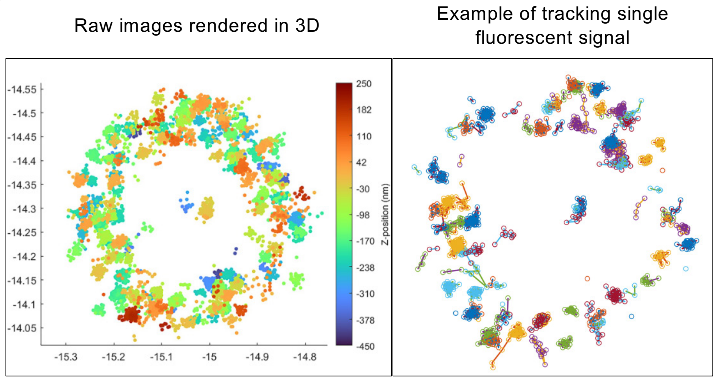

c

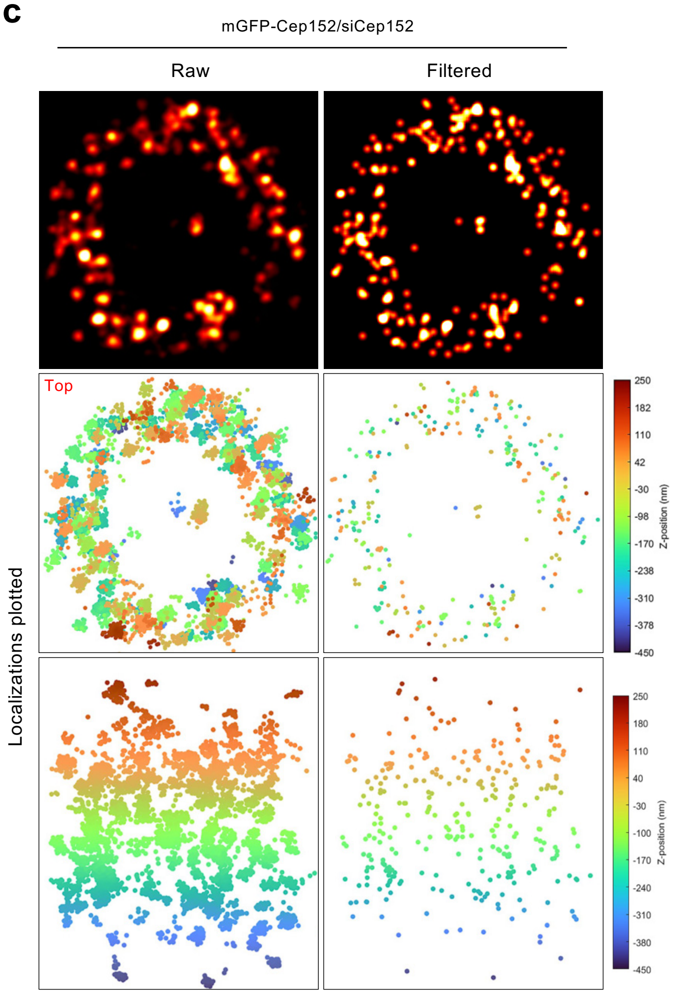

d

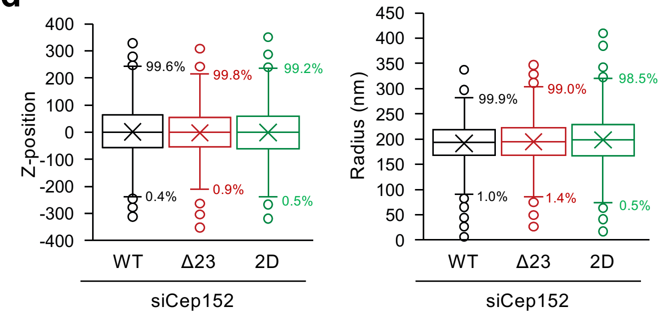

e

mGFP-Cep152 fluorescence in siCep152 cells

		Height (nm)	Mean radius (nm)	Inner radius (nm)	Outer radius (nm)	Radial width (nm)	Volume (aL)	Avg.* number	Conc. (µM)
Entire min–max range	WT	363.6 [a] ± 69.2	191.8 ± 12.1	98.6 ± 12.1	260.4 ± 15.6	161.8 ± 18.4	66.7 ± 17.4	218.2 [a] ± 73.8	5.3 [a] ± 1.5
	Δ23	305.7 [b] ± 57.7	195.2 ± 20.9	97.4 ± 20.2	279.9 ± 34.7	182.0 ± 34.6	67.5 ± 25.3	93.5 [b] ± 40.6	2.7 [b] ± 1.8
	2D	349.7 ± 58.6	198.8 ± 28.3	109.8 ± 25.0	287.4 ± 54.1	177.6 ± 49.3	82.56 ± 47.5	108.1 [b] ± 47.5	2.7 [b] ± 1.7
5th–95th percentile of the min–max range	WT	262.4 ± 43.5	192.1 ± 12.1	132.9 ± 19.1	241.6 ± 13.2	108.7 ± 22.0	33.1 ± 6.9	178.0 [a] ± 60.2	8.6 [a] ± 2.5
	Δ23	219.0 [a] ± 61.6	198.3 ± 22.0	145.2 ± 25.6	253.3 ± 27.8	108.0 ± 23.2	29.5 ± 10.4	73.0 [b] ± 33.9	4.3 [b] ± 2.4
	2D	268.0 [b] ± 49.0	199.3 ± 28.0	139.5 ± 24.8	255.9 ± 39.7	116.5 ± 27.1	40.4 ± 18.9	88.0 [b] ± 38.8	4.2 [b] ± 2.5

* Note the average number of mGFP-Cep152 is likely undercounted due to multiple reasons including antibody labeling efficiency. $P < 0.05$ between ***a*** and ***b*** (unpaired two-tailed *t*-test). No statistical difference between ***b*** and ***b***.

f

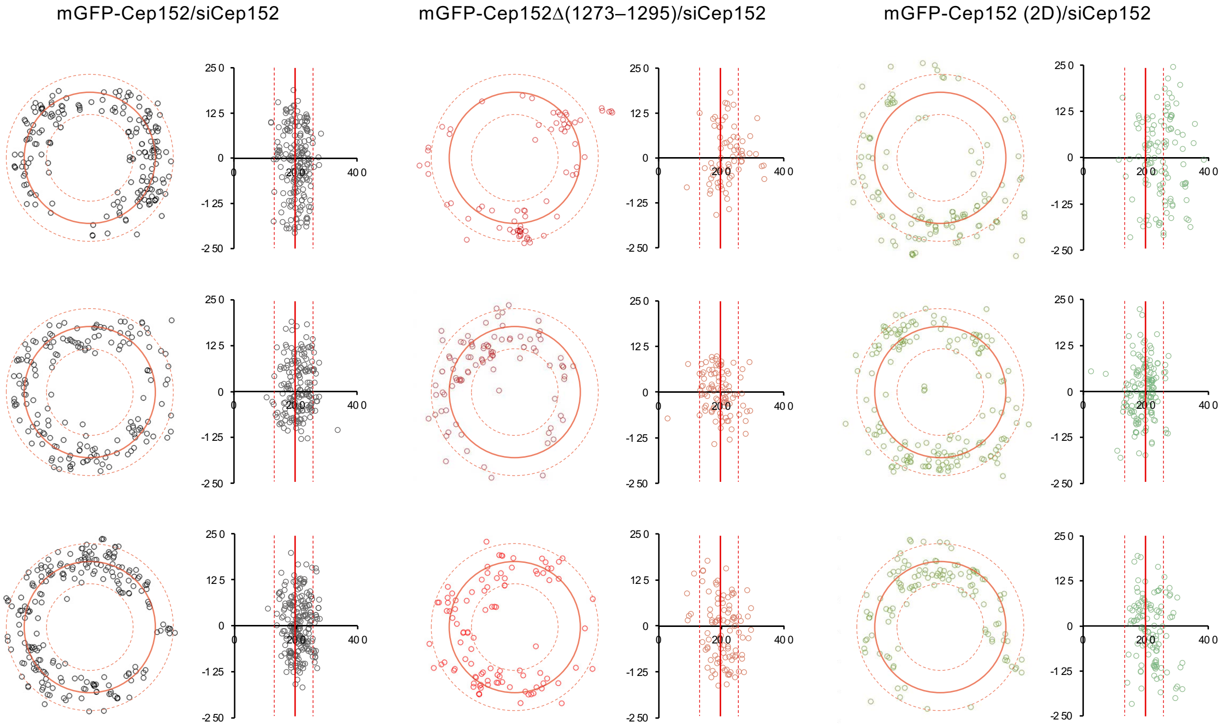
mGFP-Cep152/siCep152
mGFP-Cep152Δ(1273–1295)/siCep152
mGFP-Cep152 (2D)/siCep152
250
125
0
-125
-250
0
200
400

g

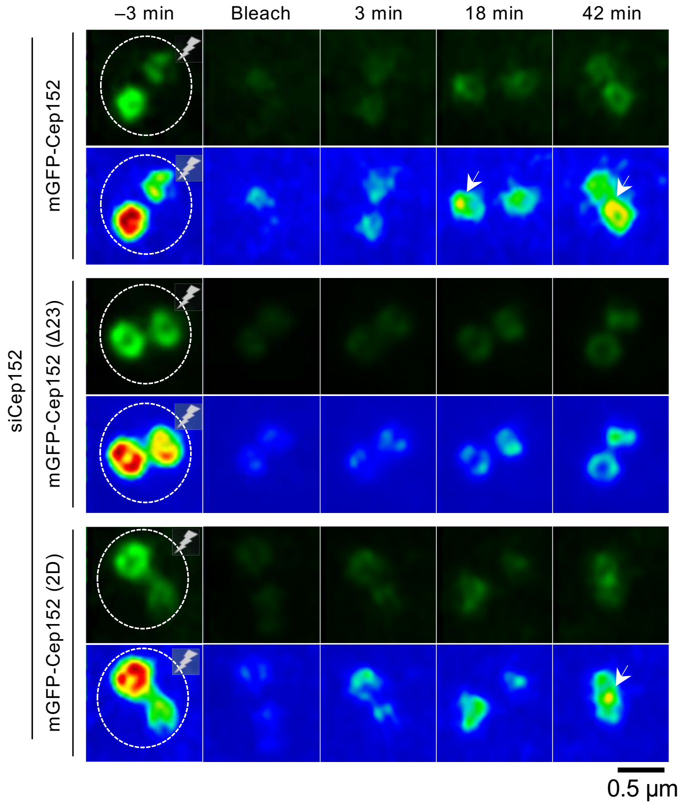
–3 min
Bleach
3 min
18 min
42 min
siCep152
mGFP-Cep152
mGFP-Cep152 (Δ23)
mGFP-Cep152 (2D)
0.5 µm

Supplementary Fig. 7. Analyses of centrosome-localized Cep152 WT and its respective Δ23 and 2D mutants. **a** Generation of U2OS cells expressing the indicated RNAi-insensitive mGFP-Cep152 constructs and depleted of endogenous Cep152 by RNAi. Immunoblotting shows the expression levels of Cep152 WT and mutants. Asterisk, a degradation product; CBB, Coomassie Brilliant Blue–stained membrane. **b, c** Images showing how MINFLUX signals were tracked and filtered to identify individual mGFP molecules. The raw 3D localizations in (**b**, left) are shown with depth coding. The image in (**b**, right) shows an example of the grouping of localizations into traces as MINFLUX repeatedly localizes each molecule. **c,** Raw (left panels) and its filtered and processed (right panels) images are provided with their localizations plotted in depth coding along the z axis. Images were filtered by removing traces with less than 5 localizations and then averaging the coordinates for each trace to obtain a single position for each trace. The resulting images are provided in Fig. 7a, left. **d–f** Analyses of MINFLUX images were carried out, with 15 images each for WT, Δ23, and 2D, obtained from three experiments. A box plot analysis in (**d**) was performed to determine the minimum and maximum values (the end of lower and upper whiskers, respectively) for each sample group. After discarding the outliers outside of the minimum and maximum values, the remaining data points were analyzed to yield (**e**) and the data shown in Fig. 7b–e (see "Statistical analysis" in the Methods for details). **f** Three representative MINFLLUX images for Cep152 WT, Δ23, and 2D are shown. The top image of each column was used to generate Fig. 7a. The vertically distributed mGFP-Cep152 signals (shown at right) obtained from all the WT (n = 15), Δ23 (n = 15), and 2D (n = 15) images were collectively analyzed to generate Fig. 7c–e after excluding outliers as determined by the analysis in (**d**). The solid red line (median of Cep152 WT) and the dotted red lines (5th–95th percentiles of Cep152 WT) are shown for easier comparison among different groups. **g** FRAP analyses for the mGFP fluorescence after depleting endogenous Cep152 by RNAi. Images were acquired every 3 minutes after photobleaching up to 42 minutes. Arrow, signals showing significant recovery. Quantified data are provided in Fig. 7f.

Supplementary Fig. 8

Supplementary Fig. 1a

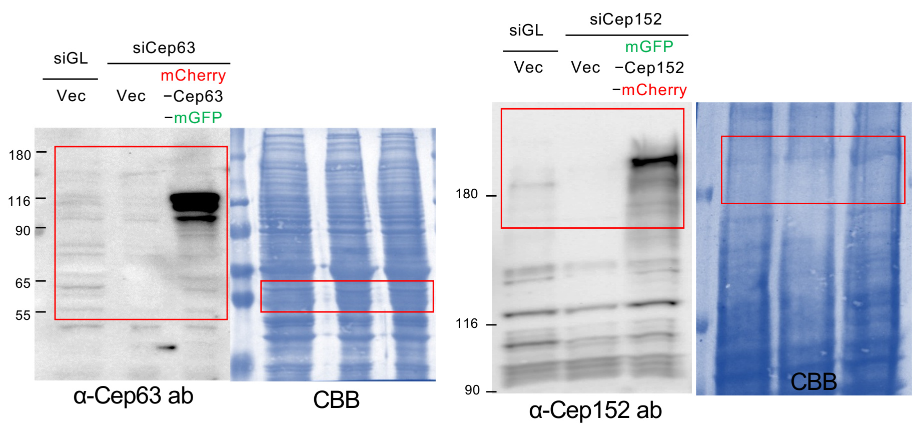

Supplementary Fig. 6a

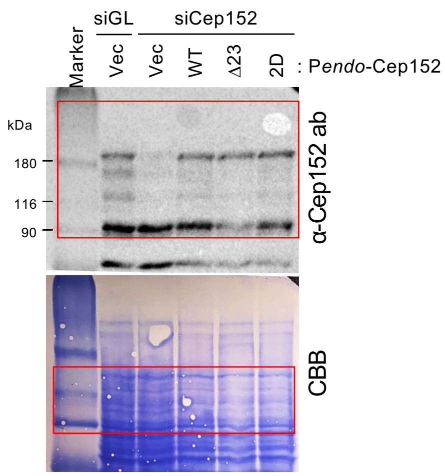

Supplementary Fig. 6g

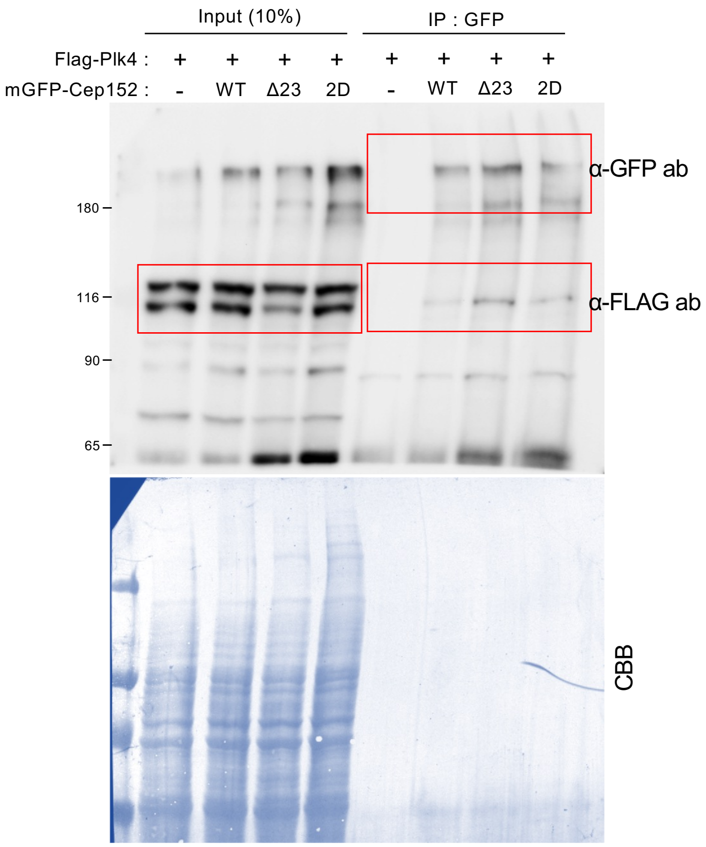

Supplementary Fig. 7a

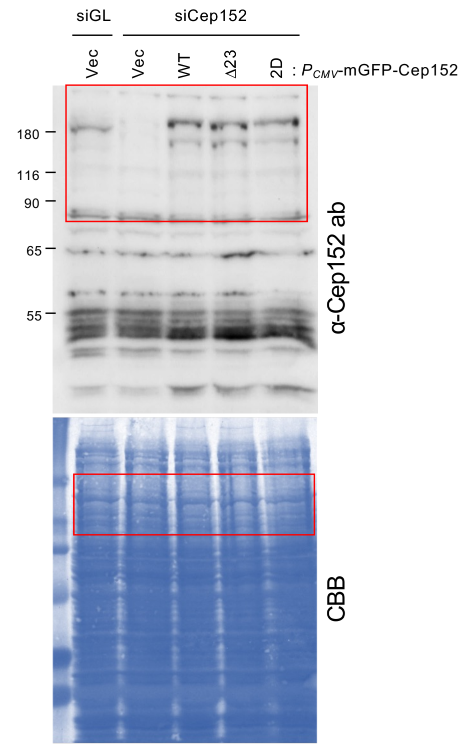

Supplementary Fig. 6n

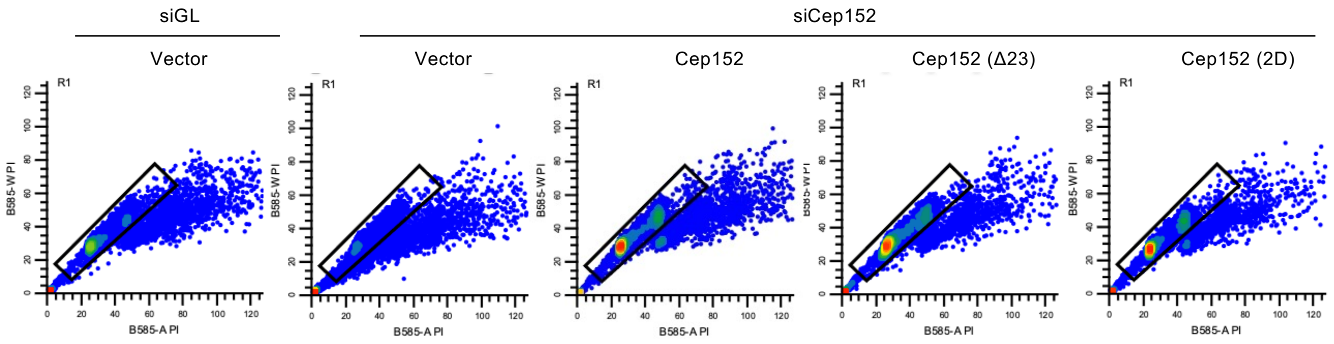

Supplementary References

1 Bauer, M., Cubizolles, F., Schmidt, A. & Nigg, E. A. Quantitative analysis of human centrosome architecture by targeted proteomics and fluorescence imaging. *EMBO J* **35**, 2152-2166 (2016). https://doi.org:10.15252/embj.201694462

2 Kim, T. S. *et al.* Molecular architecture of a cylindrical self-assembly at human centrosomes. *Nat Commun* **10**, 1151 (2019). https://doi.org:10.1038/s41467-019-08838-2

3 Elbashir, S. M. *et al.* Duplexes of 21-nucleotide RNAs mediate RNA interference in cultured mammalian cells. *Nature.* **411**, 494-498. (2001).

4 Zhao, H. *et al.* The Cep63 paralogue Deup1 enables massive de novo centriole biogenesis for vertebrate multiciliogenesis. *Nat Cell Biol.* **15**, 1434-1444. (2013).

5 Cizmecioglu, O. *et al.* Cep152 acts as a scaffold for recruitment of Plk4 and CPAP to the centrosome. *J. Cell Biol.* **191**, 731-739 (2010).

6 Kim, T.-S. *et al.* Hierarchical recruitment of Plk4 and regulation of centriole biogenesis by two centrosomal scaffolds, Cep192 and Cep152. *Proc. Natl. Acad. Sci. USA* **110**, E4849-4857 (2013).

Supplementary Table 1. Plasmid constructs used in this study

List	Protein	Residues	Expression vector	Restriction site
Mammalian expression constructs				
pKM7537	P*endo*-Cep152-*sil*	1–1654	pHR′.J-CMV-SV-puro (pKM2994)	ClaI, SalI
pKM7685	P*endo*-Cep152-*sil* Δ(1273–1295) (Δ23)	1–1654	pHR′.J-CMV-SV-puro (pKM2994)	ClaI, SalI
pKM7684	P*endo*-Cep152-*sil* (I1279D L1286D) (2D)	1–1654	pHR′.J-CMV-SV-puro (pKM2994)	ClaI, SalI
pKM7785	P_{CMV}-mGFP-Cep152-*sil*	1–1654	pHR′.J-CMV-SV-puro (pKM2994)	AscI, PmeI
pKM7786	P_{CMV}-mGFP-Cep152-*sil* Δ(1273–1295) (Δ23)	1–1654	pHR′.J-CMV-SV-puro (pKM2994)	AscI, PmeI
pKM7787	P_{CMV}-mGFP-Cep152-*sil* (I1279D L1286D) (2D)	1–1654	pHR′.J-CMV-SV-puro (pKM2994)	AscI, PmeI
pKM7788	P_{CMV}-mCherry-Cep63-*sil*-mGFP	1-541	pHR′.J-CMV-SV-puro (pKM2994)	AscI, SalI[ef]/PmeI
pKM7789	P_{CMV}-mGFP-Cep152-*sil*-mCherry	1-1654	pHR′.J-CMV-SV-puro (pKM2994)	AscI, PmeI
pKM7710	mGFP-Cep152-*sil*-mCherry	1–1654	pShuttle-CMV	BglII, NotI
pKM7713	mGFP-Cep152-*sil*-mCherry	1–1654	pAdEasy-1	Homologues recombination
pKM7737	mGFP-Cep152-*sil* Δ(1273–1295)-mCherry (Δ23)	1–1654	pShuttle-CMV	BglII, NotI
pKM7739	mGFP-Cep152-*sil* Δ(1273–1295)-mCherry (Δ23)	1–1654	pAdEasy-1	Homologues recombination
pKM7755	mGFP-Cep152-*sil*-mCherry (I1279D L1286D) (2D)	1–1654	pShuttle-CMV	BglII, NotI
pKM7756	mGFP-Cep152-*sil*-mCherry (I1279D L1286D) (2D)	1–1654	pAdEasy-1	Homologues recombination
E. coli expression constructs				
pKM5615	Cep63 (424–541)	424–541	pETDuet-1	BamHI, NotI
	Cep152 (1205–1295)	1205–1295		NdeI, Xho I
pKM6018	Cep63 (220–541)	220–541	pETDuet-1	BamHI, NotI
	Cep152 (1140–1295)	1140–1295		NdeI, Xho I
pKM5958	Cep63 (219–541)	219–541	pETDuet-1	BamHI, NotI
	Cep152 (1205–1295)	1205–1295		NdeI, Xho I
pKM5263	Cep63 (424–541)	424–541	pETDuet-1	BamHI, NotI
	Cep152 (1205–1272)	1205–1272		NdeI, Xho I
pKM5628	Cep63 (440–541)	440–541	pETDuet-1	BamHI, NotI
	Cep152 (1205–1272)	1205–1272		NdeI, Xho I
pKM7733	Cep63 (424–541)	424–541	pETDuet-1	BamHI, NotI
	Cep152 (1205–1295) (I1279D L1286D)	1205–1295		NdeI, XhoI
pKM6656	Cep63 (440–490)	440–490	pET28a	BamHI, XhoI
pKM6657	Cep63 (440–490) (L445A, A456K, L463K)	440–490	pET28a	BamHI, XhoI

pKM6658	Cep63 (440–490) (L469A, N473R, V480K)	440–490	pET28a	BamHI, XhoI

Supplementary Table 2. siRNA sequences used in this study

Target Gene	Sequence (nt positions from the start codon)	Source	Type
Luciferase	CGTACGCGGAATACTTCGA	[3]	Synthetic
Cep63	GGAGCTCATGAAACAGATT (78–96)	[4]	Synthetic
Cep152	GCGGATCCAACTGGAAATCTA (3099–3119)	[5]	Synthetic

Supplementary Table 3. Antibodies used in this study

Antibodies	Species	Cat #	Source	Exp. dilution
Primary antibodies				
Anti-Cep152 (491–810)	Rabbit	Lab supply	[6]	1:1000 (IB) 1:100 (IF)
Anti-Cep192 (1–647)	Rabbit	Lab supply	[6]	1:100 (IF)
Anti-Plk4 (580–970)	Rabbit	Lab supply	[6]	1:200 (IF)
Anti-CP110	Rabbit	12780-1-AP	Proteintech	1:2000 (IF)
FluoTag-Q anti-GFP Alexa Fluor 647	Alpaca	N0301-AF647	NanoTag Biotechnologies	1:100 (MINFLUX)
Anti-GFP	Mouse	SC-9996	Santa Cruz Biotechnology	2.5 μg (IP)
Anti-FLAG	Rabbit	F7425	Millipore sigma	0.5 μg/mL (IB)
Secondary antibodies (Immunofluorescence)				
Anti-rabbit IgG Alexa Fluor 647-conjugated	Donkey	A-31573	Invitrogen	1:300 (IF)
Anti-rabbit IgG Alexa Fluor 594-conjugated	Donkey	A-21207	Invitrogen	1:300 (IF)
Anti-rabbit IgG Alexa Fluor 488-conjugated	Donkey	A-21206	Invitrogen	1:300 (IF)
Anti-rabbit IgG Alexa Fluor 405-conjugated	Goat	A-31556	Invitrogen	1:300 (IF)
Secondary antibodies (Immunoblot)				
Anti-rabbit IgG HRP-conjugated	Donkey	NA9340	GE Healthcare	1:3000 (IB)
Anti-GFP HRP conjugated	Mouse	SC-9996 HRP	Santa Cruz Biotechnology	1:3000 (IB)

Supplementary Table 4. Data collection and structure refinement statistics

PDB	7W91
Space group	*P*1
Unit cell dimensions	
a, b, c (Å)	80.62, 81.11, 80.85
α, β, γ (°)	65.33, 60.07, 60.20
Wavelength (Å)	0.97927
Resolution (Å)	50.0-3.30 (3.36−3.30)[a]
R_{sym}[b]	11.2 (18.7)
$I/\sigma(I)$	17.9 (4.7)
Completeness (%)	90.4 (73.2)
Redundancy	2.9
Refinement	
Resolution (Å)	50.0−3.3
Number of reflections	37120
R_{work}[c] / R_{free}	22.9 / 26.1
Number of atoms	5084
R.m.s deviations	
Bond lengths (Å)	0.003
Bond angles (°)	0.480
Ramachandran plot (%)	
Most favored region	99.3
Additionally allowed region	0.7
Average B-values (Å^2)	57.9

[a]The numbers in parentheses are statistics from the highest-resolution shell.
[b]$R_{sym} = \Sigma |I_{obs} - I_{avg}| / I_{obs}$, where I_{obs} is the observed intensity of individual reflection and I_{avg} is the average over symmetry equivalents.
[c]$R_{work} = \Sigma ||F_o| - |F_c|| / \Sigma |F_o|$, where $|F_o|$ and $|F_c|$ are the observed and calculated structure factor amplitudes, respectively. R_{free} was calculated with 9.8% of the data.

Supplementary Movie 1.
3D reconstruction of the SAXS envelopes shown in Fig. 2a and Fig. 3e.

Supplementary Movie 2.
3D reconstruction of the SAXS envelopes shown in Supplementary Fig. 2e, f, h.

Supplementary Movie 3.
3D reconstruction of the images shown in Fig. 7a.
