# Supplementary material for: Architectural basis for cylindrical self-assembly governing Plk4-mediated centriole duplication in human cells
Source: Commun Biol. 2023 Jul 11;6:712. doi: 10.1038/s42003-023-05067-8 (PMC10336005; doi:10.1038/s42003-023-05067-8)
Supplement: Supplementary file 3 — Description of Additional Supplementary Files [file 42003_2023_5067_MOESM3_ESM.pdf]

Description of Additional Supplementary Files

File name: Supplementary Video 1
Description: 3D reconstruction of the SAXS envelopes shown in Fig. 2a and Fig. 3e.

File name: Supplementary Video 2
Description: 3D reconstruction of the SAXS envelopes shown in Supplementary Fig. 2e, f, h.

File name: Supplementary Video 3
Description: 3D reconstruction of the images shown in Fig. 7a.

File name: Supplementary Data 1
Description: The source data for Figs. 1b, 4b, 5b, 5c, 6a, 6c–f, 7b, 7e, and 7f and Supplementary Figs. 6c, 6d, 6f, 6i, 6j, 6l, 6m, 7d, and 7e
